# Loss of Tiparp Results in Aberrant Layering of the Cerebral Cortex

**DOI:** 10.1523/ENEURO.0239-19.2019

**Published:** 2019-11-22

**Authors:** Giulia Grimaldi, Barbora Vagaska, Oleksandr Ievglevskyi, Elena Kondratskaya, Joel C. Glover, Jason Matthews

**Affiliations:** 1 Institute of Basic Medical Sciences, University of Oslo, Oslo 0372, Norway; 2 Department of Pharmacology and Toxicology, University of Toronto, Toronto, Ontario M5S 1A8, Canada

**Keywords:** cortex, cortex layering, mono-ADP ribosylation, PARP, post-translational modification, Tiparp

## Abstract

2,3,7,8-tetrachlorodibenzo-p-dioxin (TCDD)-inducible poly-ADP-ribose polymerase (TIPARP) is an enzyme that adds a single ADP-ribose moiety to itself or other proteins. Tiparp is highly expressed in the brain; however, its function in this organ is unknown. Here, we used *Tiparp^–/–^* mice to determine Tiparp’s role in the development of the prefrontal cortex. Loss of Tiparp resulted in an aberrant organization of the mouse cortex, where the upper layers presented increased cell density in the knock-out mice compared with wild type. Tiparp loss predominantly affected the correct distribution and number of GABAergic neurons. Furthermore, neural progenitor cell proliferation was significantly reduced. Neural stem cells (NSCs) derived from *Tiparp^–/–^* mice showed a slower rate of migration. Cytoskeletal components, such as α-tubulin are key regulators of neuronal differentiation and cortical development. α-tubulin mono-ADP ribosylation (MAR) levels were reduced in *Tiparp^–/–^* cells, suggesting that Tiparp plays a role in the MAR of α-tubulin. Despite the mild phenotype presented by *Tiparp^–/–^* mice, our findings reveal an important function for Tiparp and MAR in the correct development of the cortex. Unravelling Tiparp’s role in the cortex, could pave the way to a better understanding of a wide spectrum of neurological diseases which are known to have increased expression of *TIPARP*.

## Abstract (precis)

2,3,7,8-tetrachlorodibenzo-p-dioxin (TCDD)-inducible poly-ADP-ribose polymerase (TIPARP) is an enzyme that adds a single ADP-ribose moiety to itself or other proteins. Tiparp is highly expressed in the brain; however, its function in this organ is unknown. Here, we used *Tiparp^–/–^* mice to determine Tiparp’s role in the development of the prefrontal cortex.

## Significance Statement

2,3,7,8-tetrachlorodibenzo-p-dioxin (TCDD)-inducible poly-ADP-ribose polymerase (TIPARP) is an enzyme which adds a single ADP-ribose moiety to itself or other proteins and is highly expressed in the brain. However, its function in this organ remains unknown. Here, we show that Tiparp affects neural progenitor cell proliferation and migration, and its loss leads to aberrant organization of the mouse cortex, predominantly by disrupting the correct distribution and number of GABAergic neurons. Cytoskeletal components, such as α-tubulin, are key regulators of neuronal differentiation and cortical development, here, we show that Tiparp is part of a complex that interacts with α-tubulin and plays a role in its mono-ADP ribosylation (MAR). Despite the mild phenotype presented by the *Tiparp* knock-out mouse our findings reveal an important function of MAR by this enzyme in the correct development of the cortex.

## Introduction

2,3,7,8-tetrachlorodibenzo-p-dioxin (TCDD)-inducible poly-ADP-ribose polymerase (TIPARP; also known as, PARP7/ARTD14/RM1) is expressed in many different tissues, including liver, heart, spleen, brain, and reproductive organs (Ma et al., 2001). TIPARP is one of 17 members of the PARP family that have diverse cellular functions but share a conserved catalytic PARP domain. They use nicotinamide adenine dinucleotide (NAD^+^) as a substrate to transfer one or more molecules of ADP-ribose to specific amino acid residues on target proteins. TIPARP mono-ADP ribosylates itself and other proteins including, TANK-binding protein 1 (TBK1) and the aryl hydrocarbon receptor (AHR; MacPherson et al., 2013; Ahmed et al., 2015). TIPARP regulates the pluripotency of embryonic stem cells, mediates Type I interferon-dependent antiviral responses, regulates autophagy and acts as a transcriptional repressor (MacPherson et al., 2013; Roper et al., 2014; Yamada et al., 2016; Han et al., 2018). It contains an N-terminus, followed by a CCCH-zinc finger domain, a WWE (tryptophan–tryptophan–glutamate) domain, and the conserved PARP catalytic domain. Within the N-terminus there is a short nuclear localization sequence which, along with the zinc finger domain, ensures that TIPARP is predominantly found in the nucleus; however, localization of TIPARP varies among cell lines and during different stages of the cell cycle (Vyas et al., 2013; Gomez et al., 2018). In HeLa cells, TIPARP has a diffuse cytoplasmic localization during mitosis, but it forms nuclear foci during interphase (Vyas et al., 2013). Moreover, in response to viral infection, reactive oxygen species (ROS), and mitochondrial damage, TIPARP translocates from the nucleus to the cytosol (Kozaki et al., 2017).

AHR is a ligand responsive transcription factor predominantly studied for its ability to mediate toxic responses of environmental pollutants, such as TCDD (Fernandez-Salguero et al., 1996). However, it is activated by numerous endogenous ligands and plays important roles in immune function, inflammation, stem cell differentiation, neurologic development and behavior (Stockinger et al., 2014). It has previously been reported that TIPARP functions as part of a negative feedback loop regulating AHR activity and that *Tiparp^–/–^* mice exhibit an increased sensitivity to dioxin-induced toxicities primarily due to increased AHR activity (Hutin et al., 2018).

The effects of TCDD and AHR in neurons, brain and cognitive functions has been investigated in numerous studies (Powers et al., 2005; Hood et al., 2006; Nishijo et al., 2007; Collins et al., 2008; Lin et al., 2008; Kimura et al., 2017). TCDD exposure in rodents has been reported to perturb the dendritic morphology of neurons (Kimura et al., 2015), cortical layer formation (Mitsuhashi et al., 2010), and neurogenesis in cerebellar granule cells (Collins et al., 2008). Treated rodents also exhibit a distinct decrease in behavioral flexibility, associate learning, memory, neuronal response to stimuli, locomotor activity and social behavior as well as increased anxiety. The majority of these effects are mediated by AHR, which has an important role in regulating developmental processes required for higher brain function in adulthood (Kimura and Tohyama, 2017). Recently, TCDD treatment was reported to promote migration of astrocytes in an Ahr-dependent manner and affect GABAergic neurons resulting in their altered distribution in the medial prefrontal cortex, although the mechanism of action remains elusive (Nguyen et al., 2013; Chen et al., 2019). In *Caenorhabditis elegans*, *ahr-1*, the orthologue of *AHR*, is implicated in specifying GABAergic neuron cell fate (Huang et al., 2004).

In humans, children exposed to TCDD and TCDD-like environmental pollutants before birth have impaired higher cognitive functions, which is supported by another report linking exposure to AHR activating poly aromatic hydrocarbons with intelligence in children (Seegal, 1996; Perera et al., 2009).

Increased Tiparp expression was observed in rat brains during the developmental formation of the neural network and during long-term potentiation (LTP) in adults (Matsuo et al., 2000). This suggests that TIPARP functions in brain development and in neuronal plasticity in adulthood, and may represent a key enzyme involved in molecular cascades that lead to structural changes in synaptic connections. Moreover, TIPARP was identified as a highly upregulated protein following trace fear conditioning and in neurologic disorders, such as epilepsy (Steenland, 2010; Dachet et al., 2015). In an integrated multi-cohort transcriptional meta-analysis of neurodegenerative diseases, TIPARP was shown to be strongly upregulated independently of age (Li et al., 2014). Despite several reports identifying the *TIPARP* gene or its product as being elevated in neuronal diseases, there are no studies that investigate the physiologic role of Tiparp in the brain. Unravelling its potential role in the brain could pave the way to better understanding a wide spectrum of neurologic diseases. Herein, we investigated the effects of loss of Tiparp in the mouse cortex.

## Materials and Methods

### Animals

All experiments were approved by the Norwegian Animal Research authority (FOTS ID 10420). Mice were housed in a 12/12 h light/dark cycle and had access to food and water *ad libitum*. The *Tiparp^–/–^* mice where exon 3 of the Tiparp gene was removed were generated in C57BL/6N strain. A full description of the generation of these mice has been previously described (Hutin et al., 2018b). The animal colony was maintained by breeding heterozygotes and thus strain-specific wild-type (*Tiparp* ^+/+^) control mice or *Tiparp^–/–^* mice were used in all experiments. Mice of either sex and wherever possible same sex littermates were used in these studies.

### RNA isolation and qPCR

Mice were euthanized by open-drop exposure to isoflurane and cervical dislocation. The brains were extracted in ice-cold PBS, various brain regions were dissected and flash frozen in liquid nitrogen. The tissue was then homogenized in TRIzol reagent (Thermo Fisher Scientific) and total RNA and RNA from mouse neural stem cells (mNSCs) were isolated with Aurum Total RNA Mini kit (Bio-Rad) according to the manufacturer’s instructions. All RNA was reverse transcribed using High-Capacity cDNA Reverse Transcription kit (Thermo Fisher Scientific). Transcript levels were quantified by qPCR using KAPA SYBR Fast Universal kit (Sigma-Aldrich). The amplified genes were normalized to tata-binding protein (*Tbp*). Primer sequences are provided in Table 1.

**Table 1.** Primer sequences of the primers used for qPCR

Gene name	Forward primer sequence	Reverse primer sequence
*Blbp*	GGACACAATGCACATTCAAGAAC	CCGAACCACAGACTTACAGTTT
*Cyp1a1*	CGTTATGACCATGATGACCAAGA	TCCCCAAACTCATTGCTCAGAT
*Dcx*	CCTTGATGGAAAGCAGGTCAC	GGCCTGGGCTTTTAGCAGAT
*Gfap*	CAGATCCGAGGGGGCAAAAG	TGGCAGGGCTCCATTTTCAA
*Glast1*	ACCAAAAGCAACGGAGAAGAG	GGCATTCCGAAACAGGTAACTC
*Nestin*	CCCTGAAGTCGAGGAGCTG	CTGCTGCACCTCTAAGCGA
*Nfh*	GCCGCTTACAGAAAGCTCCT	ATGTGCGTGGATATGGAGGG
*Ng2*	GGGCTGTGCTGTCTGTTGA	TGATTCCCTTCAGGTAAGGCA
*Olig1*	TCTTCCACCGCATCCCTTCT	CCGAGTAGGGTAGGATAACTTCG
*Pax6*	TACCAGTGTCTACCAGCCAAT	TGCACGAGTATGAGGAGGTCT
*Pdgfr*	AGAGTTACACGTTTGAGCTGTC	GTCCCTCCACGGTACTCCT
*Sox2*	ACAGAGAAAACCTGAGGGCG	GAAGCGCCTAACGTACCACT
*TBP*	GCACAGGAGCCAAGAGTGAA	TAGCTGGGAAGCCCAACTTC
*Tiparp*	ACATCACACCGTATTGCCCT	GCCCAAAAGTCTTGTCCTCCAT
*Tubb3*	TGAGGCCTCCTCTCACAAGTA	CCGCACGACATCTAGGACTG

### Immunohistochemistry

Mice were euthanized as described above and the brains were dissected in ice-cold PBS and then fixed overnight in 4% paraformaldehyde (PFA). Fixed brains were washed with PBS and incubated overnight in 30% (w/v) sucrose before embedding in Optimal Cutting Temperature compound (OCT). All mouse brain tissue was cryo-sectioned into 15-μm-thick sections, which were air dried before use. Unless noted otherwise, sagittal section from comparable lateral regions within a third from the midline were used.

For Nissl staining, sections were placed in 0.1% cresyl violet solution for 10 min at 37° before rinsing them in distilled water and then differentiated in 95% ethyl alcohol for 15 min. The sections were then dehydrated in 100% alcohol twice for 5 min. The sections were cleared in xylene twice for 5 min before mounting.

mNSCs were grown on poly-D-lysine coated glass cover slips, whereas COS-1 cells were grown on non-coated cover slips. Both cell types were fixed for 15 min in 4% PFA before proceeding with immunofluorescence staining.

For immunofluorescence sections or cells were rinsed in PBS then blocked for 45 min in in 0.1% Triton X-100, 10% BSA in PBS at room temperature. Sections were then incubated overnight at 4°C with primary antibodies. They were washed in PBS and incubated at room temperature for 1 h with species-appropriate Alexa Fluor or cyanine dye conjugated secondary antibodies and Hoechst 33258 (Sigma) and mounted with Vectashield anti-fading mounting medium (Vectorlabs). The antibodies used are provided in Table 2. Images were obtained with a Zeiss LSM 710 confocal or Zeiss Axio Observer microscope. Immunohistochemistry images were analyzed with CellProfiler 3.0 software (www.cellprofiler.org; Broad Institute; Carpenter et al., 2006) and bin analysis, where the thickness of the cortex was divided into 40 equal intervals, was done with KNIME Analytics Platform (www.knime.com; Berthold et al., 2009)

**Table 2.** List of primary and secondary antibodies used

Antibody	RR ID	Target antigen	Manufacturer	Catalog number	**Host**	**Clone**	**Dilution**
GFP	AB_300798	GFP	Abcam	ab13970	chicken	polyclonal	1:3000 (IHC)
GFP	AB_303395	GFP	Abcam	ab290	rabbit	polyclonal	1:1000 (WB)
Parvalbumin	AB_2174013	Ca^+2^-binding site/specifically stains the Ca^+2^-bound form of parvalbumin	Millipore	MAB1572	mouse	monoclonal, IgG1, clone PARV-19	1:500 (IHC)
Calbindin	AB_10000340	Calbindin D-28k	Swant	CB 38	rabbit	polyclonal	1:1000 (IHC)
Calretinin	AB_10000321	Calretinin human	Swant	7699/3H	rabbit	polyclonal	1:200 (IHC)
NeuN-biotin conjugated	AB_2298772	Neuron-specific nuclear protein/FOX3/RBFOX3	Millipore	MAB377B	mouse	monoclonal IgG1, clone A60	1:500 (IHC)
Biotinylated Wisteria Floribunda agglutinin (WFA)	N/A	N-acetylgalactosamine (GalNAc)	Sigma	L1766	N/A	N/A	1:100 (IHC)
GFAP	AB_10013382	Glial fibrillary acidic protein	Dako	Z0334	rabbit	polyclonal	1:400 (IHC)
TUBB3	AB_262133	Tubulin, βIII	Sigma	T2200	rabbit	polyclonal	1:500 (IHC)
SOX2	N/A	Transcription factor SOX-2	Stem Cell Tech	60055	rabbit	polyclonal	1:100 (IHC)
Nestin/radial glial marker	AB_531887	Nestin	DSHB	RC2	mouse	monoclonal, IgM	1:10 (IHC)
Vimentin	AB_531888	Vimentin	DSHB	3CB2	mouse	monoclonal, IgM	1:10 (IHC)
Pax6	AB_291612	Pax6	Covance Research Products	PRB-278P	rabbit	polyclonal	1:100 (IHC)
Pax6	AB_528427	Pax6	DSHB	PAX6	mouse	monoclonal IgG1	1:10 (IHC)
CNP/Rip	AB_531796	2',3'-cyclic-nucleotide 3'-phosphodiesterase (CNP)	DSHB	Rip	mouse	monoclonal, IgG1	1:100 (IHC)
Synapsin ½	AB_1106784	Synapsin 1/2	Synaptic Systems	106 004	guinea pig	polyclonal	1:200 (IHC)
Ctip2	AB_2064130	BCL11B	Abcam	ab18465	rat	polyclonal	1:500 (IHC)
Satb2	AB_882455	SATB2	Abcam	ab51502	mouse	monoclonal, IgG1	1:200 (IHC)
α-Tubulin	AB_477579	α-Tubulin	Sigma	T5168	mouse	monoclonal, IgG1, clone B-5-1-2	1:500 (IHC), 1:10000 (WB), 2.5μg/rxn (IP)
β-Actin	AB_476697	b-actin	Sigma	A2228	mouse	monoclonal, IgG1, clone AC-74	1:10000 (WB)
pH3	AB_310177	Phospho-histone H3 (Ser10)	Millipore	06-570	rabbit	polyclonal	1:500 (IHC)
Acetylated α-tubulin	AB_477585	α-Tubulin, acetylated (Lys-40)	Sigma	T6793	mouse	monoclonal, IgG2b, clone 6-11B-1	1:1000 (WB)
Anti-mono-ADP-ribose binding reagent	AB_2665469	Mono ADP-ribose	Millipore	MABE1076	E.coli	monoclonal	1:1000 (WB)
Secondary antibody	RR ID	Conjugated	Manufacturer	Catalog number	**Host**		
Anti-mouse IgG	AB_330924	HRP	Cell Signaling	7076S	horse		
Anti-rabbit IgG	AB_2099233	HRP	Cell Signaling	7074S	goat		
Anti-chicken IgY	AB_2534096	Alexa Fluor 488	Thermo Fisher	A11039	goat		
Anti-guinea Pig	AB_2337438	Alexa Fluor 488	Jackson ImmunoResearch	106-545-003	goat		
Anti-mouse IgG	AB_2338685	Cy3	Jackson ImmunoResearch	115-165-062	goat		
Anti-Mouse IgG	AB_2340846	Alexa Fluor 488	Jackson ImmunoResearch	715-545-150	donkey		
Anti-mouse IgM, μ chain specific	AB_2340844	Alexa Fluor 488	Jackson ImmunoResearch	715-546-020	donkey		
Anti-rabbit IgG	AB_2307443	Cy3	Jackson ImmunoResearch	711-165-152	donkey		
Anti-rabbit IgG	AB_143165	Alexa Fluor 488	Thermo Fisher	A11008	goat		
Anti-rat IgG	AB_2535794	Alexa Fluor 488	Thermo Fisher	A-21208	donkey		
Streptavidin	AB_2337246	Cy2	Jackson ImmunoResearch	016-220-084			
Streptavidin	AB_2337244	Cy3	Jackson ImmunoResearch	016-160-084			

### Electrophysiology

For whole-cell patch-clamp experiments, mice (P14–P21) were euthanized as described above, and the brain rapidly removed to ice-cold oxygenated (95%O_2_/5%CO_2_) artificial CSF (ACSF; 128 mM NaCl, 4 mM KCl, 21 mM NaHCO_3_, 0.5 mM NaH_2_PO_4_, 30 mM glucose, 1 mM MgCl_2_, and 1 mM CaCl_2_; pH 7.2; 290–300 mOsm/l). Coronal brain slices of 350 μm containing the cerebral cortex at the emergence of the dorsal hippocampus were cut using a vibrating microtome (VT1000S; Leica). Slices were stored and allowed to recover at room temperature in a submerged chamber in oxygenated standard ACSF (same composition as dissection ACSF, except that CaCl_2_ was at a concentration of 2 mM) for at least 1 h before experimentation. The slices were then transferred to a submersion chamber that was perfused with the oxygenated ACSF at 31°C for whole-cell recording. The cortex primary neurons were visually identified by their pyramidal shape and regular spikes with an up-right infrared-differential interference contrast (IR-DIC) microscope (Olympus BX51WI) and captured with a CoolSNAP EZ with SSD ICX285 (Photometrics) video camera. Spontaneous IPSCs and EPSCs were recorded by using patch clamp technique in a whole-cell configuration. Patch electrodes were fabricated from borosilicate glass capillaries with an outer diameter of 1.5 mm (Sutter Instruments) using an automated puller (PC-10; Narishige). The internal pipette solution for studying excitatory synapses contained 130 mM K-methylsulfate, 5 mM NaCl, 1 mM CaCl2, 20 mM HEPES, 0.5 mM EGTA, 3 mM Mg-ATP, and 0.4 mM Na_3_GTP-GTP (Devlin et al., 2015). The solution for studying inhibitory synapses was composed of 135 mM Cs-MeSO_4_, 10 mM CsCl, 10 mM HEPES, 0.5 mM EGTA, and 4 mM Mg-ATP. Pipette solutions were freshly made and filtered (0.1 μm), osmolarity was 295–305 mOsmol, and pipette resistance was 5–7 MΩ.

To separate IPSCs and EPSCs (typically throughout the entire experiment), different holding potentials were applied (Vh = 0 and Vh = −60 or −70 for IPSCs and EPSCs, respectively. All recordings were performed using a MultiClamp 700B amplifier (Molecular Devices). Recordings and pre-processing of data were made with WinWCP (University of Strathclyde). The signals were typically low-pass filtered with a corner frequency (−3 dB) of 3 kHz and sampled at 6 kHz by DigiData 1322A (Molecular Devices).

Data analysis was performed with p-CLAMP 10 (Molecular Devices) and OriginLab 8 (OriginLab Corp.). sPSCs were detected with a threshold of 5–7 pA, depending on the noise level via template-based algorithm and by visual verification. Analyses of the kinetic properties of sPSCs included only well separated (interevent intervals >40 ms), monophasic PSCs which appeared to rise in a monotonic fashion without visible deviation of the rising phase and which decayed exponentially. The rise and decay time courses of individual PSCs were estimated by curve fitting with the exponential function:

$$A(t)=A_{0}e^{-\frac{t}{\tau }},$$

where A(t) is the current as a function of time, A_0_ is the maximum amplitude of the event (determined as the maximum current of the selected event/response), τ is the time constant, τ_RISE_ for event’s rise phase and τ_DECAY_ for decay phase

### Differentiation of mNSCs line

Primary mNSC lines isolated from dissected forebrain of E14.5 embryos from *Tiparp* ^+/+^ and *Tiparp^–/–^*mice were established and cultured as described previously (Pollard, 2013). In brief, the forebrain was dissected out in dissection solution (137 mM NaCl, 5.4 mM KCl, 0.17 mM Na_2_HPO_4_, and 0.22 mM KH_2_HPO_4_) and the meninges were removed. Each forebrain was kept separately in 1ml of Accutase and put at 37°C for 30 min and triturated with a pipette half way through the incubation. The cells were then triturated again to obtain an almost a single cell suspension in 1 ml of culture medium [DMEM/F12 supplemented with 1% penicillin/streptomycin (P/S), 2% B27, 10 ng/ml EGF, 10 ng/ml FGF, and 2.5 U/ml heparin] and centrifuged 100 × *g* for 4 min. The pellet was then resuspended in 1ml of culture medium and the non-digested tissue was left to settle by gravity for about 1 min. Cell suspension was then plated in a well of a 12-well plate. The cells were cultured as a monolayer with laminin. Differentiation of the mNSCs in the three lineages was done as previously described (Conti et al., 2005; Glaser et al., 2007; Pollard, 2013). Briefly, for neuronal differentiation cells were placed in culture medium lacking EGF for a week before changing to complete neurobasal medium (neurobasal medium containing 1% P/S, 2% B27, and 1% GlutaMAX). Experiments were done after a total of 14 d of differentiation and were maintained in complete neurobasal medium supplemented with 2 ng/ml BDNF (Peprotech). To differentiate the cells into astrocytes they were placed in DMEM containing 1% GlutaMAX, 1% P/S, and 20% fetal bovine serum for 7 d before starting the experiments. Lastly, for oligodendrocyte differentiation cells were cultured for 4 d in DMEM/F12 containing 1% P/S, 2% B27, 10 ng/ml FGF, 2.5 U/ml heparin, 10 ng/ml PDGFAA (Peprotech), and 10 μM forskolin. This is to support the glial precursor stage. To then induce differentiation, growth factors were withdrawn and cells were exposed to 30 μg/ml 3’3’5-triiodo-L-thyronine and 200 μM ascorbic acid (Sigma) for 4 d. All cell culture reagents were purchased from Thermofisher Scientific, unless stated otherwise. 

### Cell proliferation and cell death assays

Cell proliferation was assessed by methylene blue colorimetric assay as previously described (Pelletier et al., 1988). In brief, for each cell line and timepoint cells were seeded in five wells of a 96-well plate and fixed with 4% PFA for 15 min at room temperature at the indicated timepoints. They were then gently washed with PBS and incubated with methylene blue solution (1% w/v methylene blue in 0.1 M borate buffer; pH 9) for 30 min at room temperature. The excess dye was washed off with four rinses in 0.01 M borate buffer pH 9. The methylene blue was extracted using a 1:1 v/v of ethanol: 0.1 M HCl. The optical density was read at 650 nm with a SynergyH1 microplate reader.

Cell death was assessed by adding 1 μg/ml Hoechst 33258 to the cell culture media for 1.5 h and then adding 5 μg/ml propidium iodine and incubating for a further 30 min. The cells were then imaged with a Zeiss Axio Observer microscope and the ratio of live to dead cells calculated with CellProfiler 3.0 software (www.cellprofiler.org; Broad Institute; Carpenter et al., 2006).

Cell migration was assessed by a scratch assay modified from a previous study (Liang et al., 2007). For each cell line and timepoint, five wells of a 24-well plate each containing custom made 1 × 5 × 1 mm silicon inserts. Once the cells were confluent the insert was removed and the cells were placed back into an incubator. At the various timepoints the cells were fixed for 15 min at room temperature with 4% PFA and rinsed with PBS. The scratch was imaged with a Zeiss Axio Observer microscope by phase contrast. The images were then analyzed using the Fiji software (https://fiji.sc/; Schindelin et al., 2012).

### Cell-cycle analysis

Cell-cycle analysis was performed by DNA staining of cells followed by flow cytometry. Approximately one million cells per mNSCs line were resuspended by adding 1-ml methanol at –20°C drop by drop while vortexing. Cells were incubated for at least 1 h at –20°C, then 3 ml of PBS were added, and the cells were spun at 200 × *g* for 3 min. The cell pellet was resuspended in 500 ml of PBS containing 1.5 mg/ml of Hoechst 33258. After 30 min, the cells were strained and analyzed by flow ytometry.

### Immunoprecipitation

Mono-ADP-ribosylation (MAR) of α-tubulin was verified by immunoprecipitation as previously described (Hutin et al., 2018a). In brief, mNSCs were harvested and the cell pellet was lysed in cell lysis buffer mNSCs (200 mM NaCl, 1% NP40, and 20 mM HEPES at pH 7.4, supplemented with protease inhibitor cocktail; Sigma). Lysates were clarified by centrifugation and the supernatant was incubated for 2 h with anti- α-tubulin (Sigma) antibody and protein G Dynal beads (Thermo Fisher). The bead-antibody-protein complex was then washed three times for 5 min in wash buffer (200 mM NaCl, 0.1% NP40, and 20 mM HEPES at pH 7.4, supplemented with protease inhibitor cocktail; Sigma). The proteins were then eluted from the beads with 2× Laemmli buffer and separated by SDS–PAGE and transferred to PVDF membranes. Membranes were probed with anti-mono-ADP-ribose-binding reagent (Millipore) followed by incubation with the appropriate secondary antibody. A list of all antibodies is provided in Table 2.

COS-1 cells were transfected with 2 μg of EGFP, GFP-Tiparp, or GFP-Tiparp^H532A^ plasmids (MacPherson et al., 2013) per well of a six-well plate using Lipofectamine 2000 reagent according to the manufacturer’s instructions. The next morning, cells were harvested, protein extracts were made and the immunoprecipitation was conducted as described above.

### Statistical analyses

All data were presented as means and SEM. A two-tailed Mann–Whitney *post hoc*, a Kruskal–Wallis one-way ANOVA test followed by a Dunn’s multiple comparison, a two-way ANOVA test or Student’s *t* test were used to assess statistical significance (*p* < 0.05). *U* values for the Mann–Whitney test are reported in the Extended Data Figures 1-1, 4-1, 5-1.

**Figure 1.**
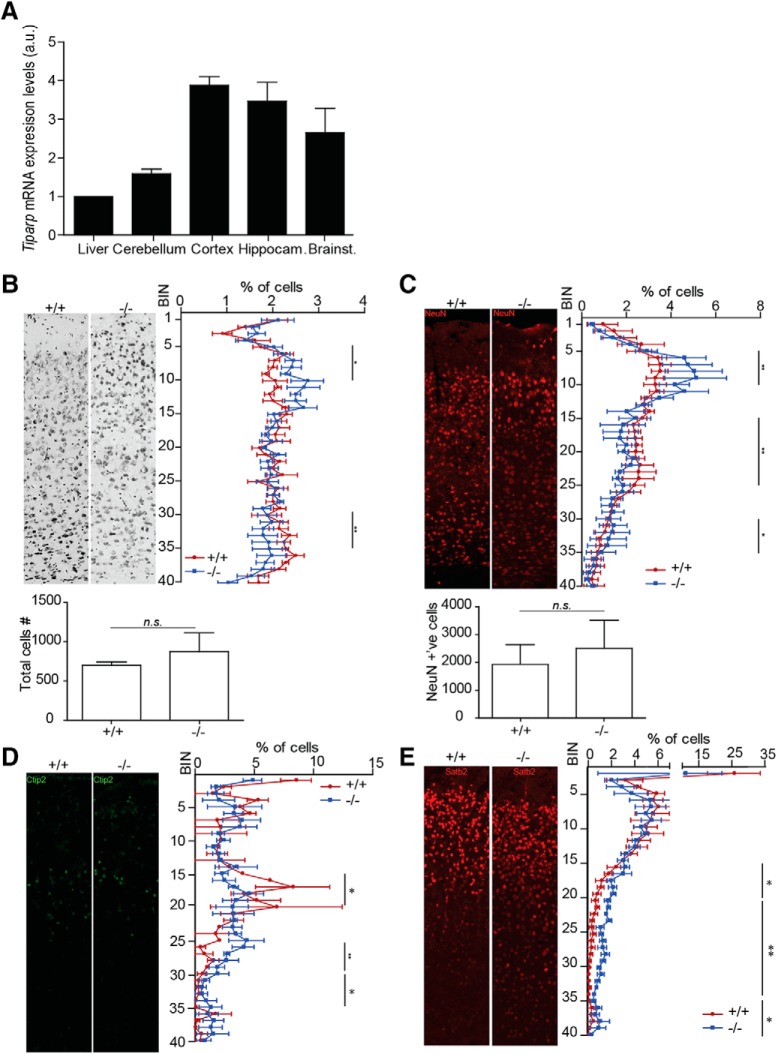
Tiparp is highly expressed in the adult mouse brain and its depletion leads to aberrant layering. ***A***, qPCR of *Tiparp* gene expression levels in cortex, cerebellum, hippocampus (hippocam.) and brainstem (brainst.), which also included the diencephalon relative to liver (*n* = 3). ***B–E***, Representative stainings of comparable sagittal sections from *Tiparp^+/+^*and *Tiparp^–/–^* mouse cortex followed by automated counting of cells and distribution analysis along the *y*-axis of the cortex. Graphs below ***B***, ***C*** show the total number of cells (***B***) and NeuN-positive cells (***C***) in the area analyzed. ***B***, Nissl staining in adult mouse (*n* = 5). ***C***, Neurons stained with NeuN in the adult mouse (*n* = 3). ***D***, Ctip2 staining of neurons in Layer V in P5 mouse (*n* = 3). ***E***, Satb2 staining predominantly of neurons in Layers II–V in P5 mouse (*n* = 3). Percentage of cells refers to the percentage of cells in a bin relative to the total number of cells in the area analyzed. The asterisk denotes significant differences between the wild type and the knock-out (*p < 0.05 or **p < 0.005), within the delimited bin range, according to a two-tailed Mann–Whitney test. *U* values for the Mann–Whitney test can be found in Extended Data Figure 1-1. Non-significance is denoted with n.s. Data are mean ± SEM. The *n* number refers to the number of mice in each group, for panels ***B–E***, multiple sections of each mouse brain were analyzed, but statistics was done per animal.

10.1523/ENEURO.0239-19.2019.f1-1 Extended Data Figure 1-1 U values for the Mann–Whitney tests in Figure 1. Download Figure 1-1, DOC file.

10.1523/ENEURO.0239-19.2019.f4-1 Extended Data Figure 4-1 U values for the Mann–Whitney tests in Figure 4. Download Figure 4-1, DOC file.

10.1523/ENEURO.0239-19.2019.f5-1 Extended Data Figure 5-1 U values for the Mann–Whitney tests in Figure 5. Download Figure 5-1, DOC file.

## Results

Tiparp mRNA expression levels were analyzed in the cortex, hippocampus, cerebellum and midbrain of two- to four-month-old C57BL/6N mice, as well as in the liver for comparison. High mRNA levels were observed in the cortex and hippocampus compared with the liver (∼4 and 3.8 times higher than in liver, respectively) as well as in the brainstem, which included the diencephalon, and the cerebellum, albeit at lower levels (2.5 and 2 times higher than in liver, respectively; Fig. 1*A*). To establish the role of Tiparp in the brain, we performed histologic analysis on the cerebral cortex of *Tiparp^–/–^* and *Tiparp^+/+^* mice at two to four months of age. The distribution of cells along the thickness of the cortex was analyzed by determining the *y*-position of each Nissl-stained cell in comparable sagittal brain sections followed by data binning. *Tiparp^–/–^* mice had a significantly higher percentage of cells in the upper layers of the cortex compared with the lower ones (Fig. 1*B*). Neuronal-specific NeuN staining suggested that the aberrant distribution of cells along the height of the cortex was due to mislocalization of neurons. *Tiparp^–/–^* mice had significantly more NeuN-positive cells in the upper layers at the expense of the lower ones compared with *Tiparp^+/+^* mice (Fig. 1*C*). Despite the differences in the cell distribution, they had comparable total cell numbers throughout the thickness of the cortex (Fig. 1*B*,*C*, lower panels).

The majority of the differences in cell distribution along the thickness of the cortex were observed around bins 5–15 (Fig. 1*A*,*B*). Because of this observation, we then investigated whether this aberrant organization of the neurons along the cortex was due to impaired migration of the neurons from the deep layers to the upper layers during development. Thus, we analyzed the expression of a specific Layer V marker, Ctip2 (Chen et al., 2008). The cortex of *Tiparp^–/–^* mice had significantly less Ctip2-positive cells in the middle of the cortex (bins 15–20), but more in the lower third of the cortex compared with *Tiparp^+/+^* ones (Fig. 1*D*). To further show disorganization of the cortex we also stained for Satb2, which is expressed in all, but predominantly, layers in II–V (Chen et al., 2008; Huang et al., 2013). The cortex of *Tiparp^+/+^* mice had more Satb2-positive neurons in bins 15–35 and significantly less in bins 35–40 compared with *Tiparp^–/–^* mice (Fig. 1*E*). Taken together, these data suggest that the loss of Tiparp causes aberrant positioning of various neuronal subtypes across all layers and the entire thickness.

**Figure 2.**
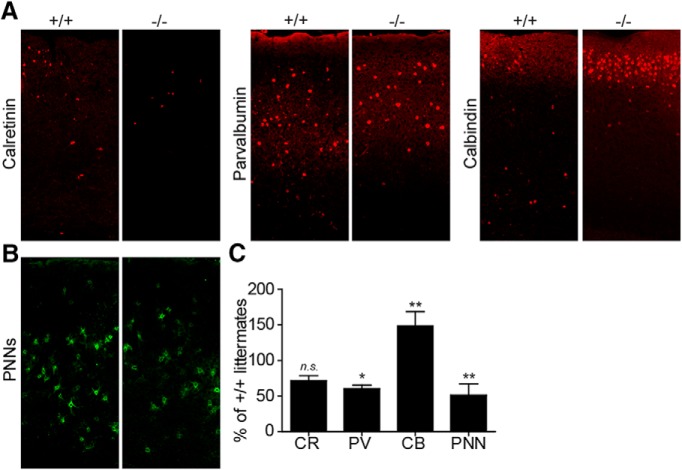
*Tiparp^–/–^* mouse cortex presents altered number of GABAergic neurons. ***A***, Fluorescent micrographs from representative staining for calretinin (CR), parvalbumin (PV), calbindin (CB), or ***B***, perineuronal nets (PNNs) in comparable sagittal sections from *Tiparp^+/+^*and *Tiparp^–/–^* adult mouse cortex. The green fluorescent staining of the PNNs was obtained by biotinylated WFA and Cy2-streptavidin. ***C***, Quantification of calretinin, parvalbumin, calbindin expressing cells, and cells surrounded by PNNs, in *Tiparp^–/–^* adult mouse cortex, expressed as percentage of the wild-type littermate (*n* = 3 mice). Data are mean ± SEM. The asterisk denotes significant differences between the wild type and the knock-out (*p < 0.05 or **p < 0.005) calculated with a two-way ANOVA test. Non-significance is denoted with n.s.

As TCDD has been previously shown to affect GABAergic neurons and TCDD increases Tiparp expression via Ahr, we investigated whether loss of Tiparp had any effects on GABAergic neurons and their distribution. The majority of these neurons in the cortex can be divided into subtypes based on the differential expression of calcium binding proteins: calbindin, calretinin or parvalbumin (Condé et al., 1994; del Río and DeFelipe, 1996; Gabbott and Bacon, 1996). Sagittal brain sections from *Tiparp^+/+^* and *Tiparp^–/–^* mice were stained for the three calcium binding proteins. *Tiparp^–/–^* mice had significantly lower (29%) calretinin and lower (40%) parvalbumin-positive neurons, but higher calbindin (148%) expressing cells compared with *Tiparp^+/+^* littermates (Fig. 2*A*,*C*). Perineuronal nets (PNNs) are extracellular matrix structures that preferentially surround parvalbumin expressing GABAergic neurons (Kosaka and Heizmann, 1989; Härtig et al., 1992). We then investigated if the decrease in parvalbumin expressing neurons correlated with a decreased formation of PNNs. *Tiparp^–/–^* mice had lower number of cells (49%) surrounded by these structures (Fig. 2*B*,*C*). However, whether loss of PNNs was due to a direct effect of Tiparp on the extracellular structures or whether this was due to a decrease in parvalbumin-positive neurons remains to be studied. Taken together, these data suggest that Tiparp affects the number and correct distribution of GABAergic neurons in the cortex.

**Figure 3.**
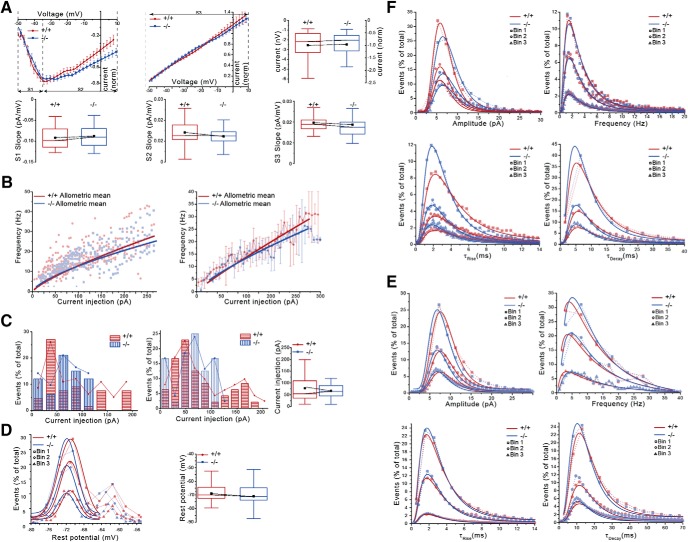
Knock-out of Tiparp does not affect the electrophysiology of cortex primary neurons. ***A***, Characterization of the currents from voltage-clamp recordings show comparable potential dependence of induced currents in the two genotypes. Averaged current−voltage curves (I-V) of inward currents (top left panel) and outward currents (top middle panel) for voltage clamp recordings from whole cells in *Tiparp^+/+^* and *Tiparp^–/–^* adult mouse cortex slices. The top right panel is a box chart representation of the maximum amplitudes of the inward currents. The bottom panel shows box chart representations of the averaged slopes of corresponded periods of the individual IV curves: the inward currents (S1 for the rise and S2 for the decay period; left and middle panel, respectively) and the entire outward currents (slope S3; right panel). These are all calculated for each recording independently. The box represents the data between the second and third quartile, the whiskers represent the range within the 1.5 interquartile range. The black square is the mean and the line within the box is the median value. Current (norm.) is the normalized current expressed in arbitrary units. ***B***, Frequencies of action potential firing during step membrane depolarization are similar for *Tiparp^+/+^* and *Tiparp^–/–^* neurons. Concatenated curves (left panel) and the averaged curves of the individual cells (right panel) for depolarization dependent frequencies of action potential firing of *Tiparp^+/+^* and *Tiparp^–/–^* neurons. The allometric function fittings f(x) = a*x^b, where a and b are parameters, were also plotted on the graphs. ***C***, Minimal current injection required to trigger enough membrane depolarization for the cells to generate an action potential firing. Distribution curves and histograms of concatenated data of current injections in *Tiparp^+/+^* and *Tiparp^–/–^* cells needed for action potential (AP) generation calculated for bin = 10 pA (left panel) and bin = 20 pA (middle panel) step size. The *y*-axis represents the fraction of cells depolarized by a given current injection (*x*-axis) sufficient for AP firing. Most of cells start AP firing when depolarized by a 40- to 80-pA current injection. The right panel is a box plot of the concatenated data that a cell generates an AP under the given minimal current injection. The data does not show normal distribution. ***D***, The left panel shows the distribution curves for concatenated data of the resting potential of the patched cells and expressed as the probability to observe a defined resting potential. The graphs represent the Gaussian curves for the data grouped by three different bin sizes. The right panel shows a box chart of the concatenated data of resting potential within the entire data range, the whiskers represent the range within the 1.5 interquartile range. The black square is the mean and the line within the box is the median value. ***E***, ***F***, Similarity of functional composition and excitability of *Tiparp^+/+^* and *Tiparp^–/–^* cortical networks: sPSCs. Whole-cell voltage clamp recordings of sIPSCs (***E***) and sEPSCs (***F***) from *Tiparp^+/+^* and *Tiparp^–/–^* adult mouse cortex slices. Concatenated data of the distribution curves for the amplitudes (top left panel); frequencies (top right panel); tau rises (bottom left panel) and tau decays (bottom right panel). The data were grouped into three bins and fitted with lognormal curves.

To determine whether Tiparp loss impaired neuronal networks and interfered with the excitability of cortical pyramidal neurons and the alternation of cortical microcircuitry, we did whole-cell patch-clamp recordings. Specifically, we investigated the characteristic current−voltage (I-V) curves from action potentials to assess the firing rate of pyramidal neurons and assessed the minimal current injection required to trigger enough membrane depolarization for the cells to generate an action potential. Both EPSCs and IPSCs were recorded. However, no significant differences were observed in any of these parameters between neurons isolated from *Tiparp^–/–^* and *Tiparp^+/+^* mice (Fig. 3*A–F*). The resting potential of the cells was also measured and it showed hyperpolarization of *Tiparp^–/–^* neurons compared with those from *Tiparp^+/+^* mice (Fig. 3*F*), suggesting alterations in function and/or number of ion channels.

**Figure 4.**
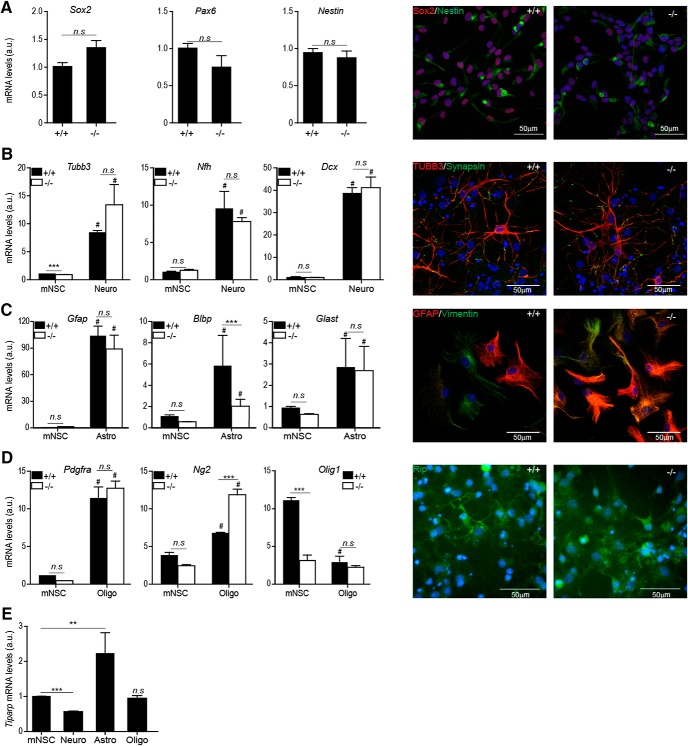
Differentiation potential of *Tiparp^–/–^* mNSCs into three lineages. ***A–D***, Gene (left panel) and protein (right panel) expression of mNSCs markers in undifferentiated cells (***A***); neuronal markers in cells differentiated toward the neuronal lineage (Neuro) for 14 d (***B***); astrocyte markers in 7-d differentiated astrocytes (Astro; ***C***) and oligodendrocyte markers in cells differentiated for 8 d toward the oligodendrocytic lineage (Oligo; ***D***). ***E***, Tiparp gene expression levels in *Tiparp^+/+^* mNSCs and in cells differentiated toward the three neural lineages, as (***p < 0.001 or **p < 0.005) between the genotypes determined by a two-tailed Mann–Whitney test (A, E; U values can be found in Extended Data Fig. 4-1) or a two-way ANOVA (***B–D***). Non-significance is denoted by n.s., and # represents statistical significance (at least *p* < 0.05) between undifferentiated and differentiated cells (***B–D***).

To elucidate the developmental mechanisms underlying the aberrant distribution of neurons in the cortex, and to understand whether the observed phenotype was due to aberrant differentiation, proliferation or migration of developing neurons, we isolated mNSCs from both *Tiparp^+/+^* and *Tiparp^–/–^* littermates. Three independent lines were established from two litters. There were no significant differences in the expression of the basic NSC markers at either mRNA level (SOX2, PAX6, Nestin) between genotypes. mNSCs derived from both genotypes expressed comparable protein levels of SOX2 and Nestin (Fig. 4*A*). Both *Tiparp^+/+^* and *Tiparp^–/–^* mNSCs differentiated into the three neural lineages: neurons, astrocytes and oligodendrocytes. Differentiation into neurons resulted in increased RNA expression levels of the neuronal markers *Tubb3*, *Nfh*, and *Dcx* compared with mNSCs. However, no significant differences in these markers were observed between genotypes (Fig. 4*B*). Astrocyte differentiation resulted in increased RNA expression levels of astrocyte-specific markers compared with undifferentiated cells (Fig. 4*C*). Of the three markers examined, we only observed significant increases in *Blbp* RNA levels between *Tiparp^–/–^* and *Tiparp^+/+^* cells. For oligodendrocyte differentiation, significant increases in *Pdgfra* and *Ng2* were observed in both genotypes compared with undifferentiated cells. The oligodendrocyte marker, *Ng2*, was significantly increased at the RNA level in *Tiparp^–/–^* compared with *Tiparp^+/+^* cells (Fig. 4*D*). *Olig1* RNA levels were significantly lower in *Tiparp^–/–^* compared with *Tiparp^+/+^* mNSCs. *Olig1* levels were significantly decreased in differentiation to oligodendrocytes in *Tiparp^+/+^* cells, but unchanged in *Tiparp^–/–^* cells. At protein level, *Tiparp^–/–^* mNSCs differentiated into the three lineages express the typical markers, suggesting that even in the absence of Tiparp the cells retain their differentiation potential (Fig. 4*B–D*). Tiparp mRNA levels were significantly downregulated during neuronal cell differentiation, whereas they were significantly upregulated on astrocyte differentiation (Fig. 4*E*). No changes were observed when cells were differentiated toward the oligodendrocyte lineage (Fig. 4*E*). This suggested that Tiparp expression is regulated during neuronal and astrocytic differentiation and it is involved in the regulation of specific genes involved in differentiation, but it is not a master regulator of neuronal stem cell differentiation.

**Figure 5.**
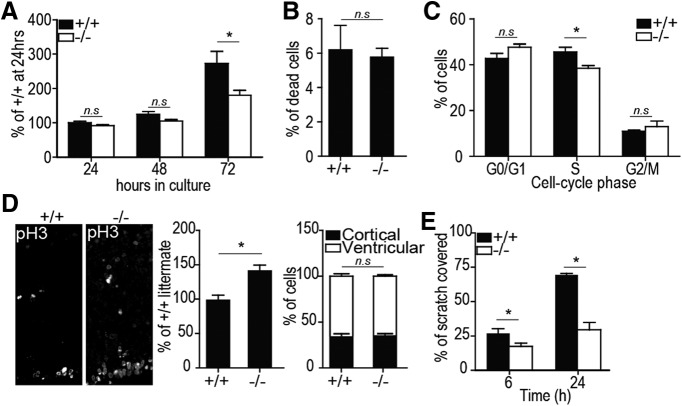
Tiparp depletion affects proliferation and migration of mNSCs. ***A***, Methylene blue assay of *Tiparp^+/+^*and *Tiparp^–/–^* mNSCs to determine proliferation rates expressed as percentage of *Tiparp^+/+^* cells 24 h after plating. ***B***, Propidium iodine and Hoescht 33342 staining of cells in both *Tiparp^+/+^*and *Tiparp^–/–^* mNSCs was used to assess cell death. The graph shows the percentage of propidium iodine-positive cells within each genotype. ***C***, *Tiparp^+/+^*and *Tiparp^–/–^* mNSCs were stained with Hoescht 33342 and subjected to flow cytometry to determine the percentage of cells in each phase of the cell cycle. All experiments were conducted on three independent mNSCs lines. ***D***, Representative image of coronal section of E17.5 *Tiparp^+/+^*and *Tiparp^–/–^* mice stained with an anti-phosphorylated histone H3 antibody (pH3), left panel and quantification of the pH3-positive cells as percentage of the wild-type littermate (middle panel) and percentage of total cells in each of the two pH3-positive cell populations determined by their position in the cortical plate or in the ventricular zone (right panel; *n* = 3 mice). ***E***, Scratch assay to assess migration capabilities of *Tiparp^+/+^* and *Tiparp^–/–^* mNSCs. Data are mean ± SEM. The asterisk denotes significant differences between the wild type and the knock-out (p < 0.05) with a two-way ANOVA (***A***, ***C***, ***D***, right panel, ***E***) or a Mann–Whitney test (***B***, ***D***, middle; U values can be found in Extended Data Fig. 5-1). Non-significance is denoted by n.s.

Next, we assessed the proliferation rate of *Tiparp^+/+^* and *Tiparp^–/–^* mNSCs by conducting a methylene blue assay. Indeed, despite having plated the same number of cells after 72 h, *Tiparp^–/–^* mNSCs had proliferated significantly less than the *Tiparp^+/+^* cells (Fig. 5*A*). Since no differences in propidium iodine staining were observed between the two genotypes, increases in cell death could not be explained by the slower growth rate of the *Tiparp^–/–^* cells (Fig. 5*B*). We then stained the cells with Hoechst 33342 and subjected them to flow cytometry to determine the percentage of cells in each phase of the cell-cycle cell. The cycle analysis revealed a significantly lower percentage of *Tiparp^–/–^* cells in the S-phase, which was accompanied by a slightly higher percentage of cells in G_0_/G_1_ and G_2_/M phases compared with *Tiparp^+/+^* cells (Fig. 5*C*). To verify this further, mNSCs in mitosis were analyzed *in vivo* in comparable coronal sections of the prefrontal cortex from *Tiparp^+/+^* or *Tiparp^–/–^* E13.5 mice stained with an anti-phosphorylated histone H3 antibody (serine 10). Histone H3 is specifically phosphorylated during mitosis where metaphase chromosomes are phosphorylated and on exit of the cell cycle a global dephosphorylation of H3 occurs (Hans and Dimitrov, 2001). As expected, prefrontal cortex in *Tiparp^–/–^* mice had significantly higher levels of phospho-histone H3-positive cells compared with their *Tiparp^+/+^* littermates. Interestingly, both the ventricular and aventricular mNSC populations were increased in the *Tiparp^–/–^* mice, but the ratio of the two populations was the same in both genotypes (Fig. 5*D*). Taken together, these data suggest a mitotic defect with abnormal cell-cycle progression resulting in a lower proliferation rate in the absence of Tiparp. Furthermore, our findings are consistent with a previous report showing that TIPARP knock-down in HeLa cells results in higher phospho-histone H3 staining and an increased length of pre-metaphase mitosis (Vyas et al., 2013).

*In vitro* migration assays of NSCs can be an indication of whether migration could also be affected *in vivo*. Thus, we assessed the effect of Tiparp loss on mNSC migration capabilities with a scratch assay. Six hours after the scratch through the center of confluent cells, *Tiparp* ^+/+^ mNSCs covered ∼28% of the scratch versus only 18% covered by the *Tiparp^–/–^* cells. This difference increased after 24 h, by which time, *Tiparp^–/–^* cells covered 31% of the scratched area while the *Tiparp* ^+/+^ mNSCs covered 73% (Fig. 5*E*). Although the molecular mechanisms underlying the aberrant distribution of neurons in the cortex remain unknown, our data suggest that cell cycle/proliferation and cell migration are two processes affected by the absence of *Tiparp* which could contribute to the observed phenotype.

**Figure 6.**
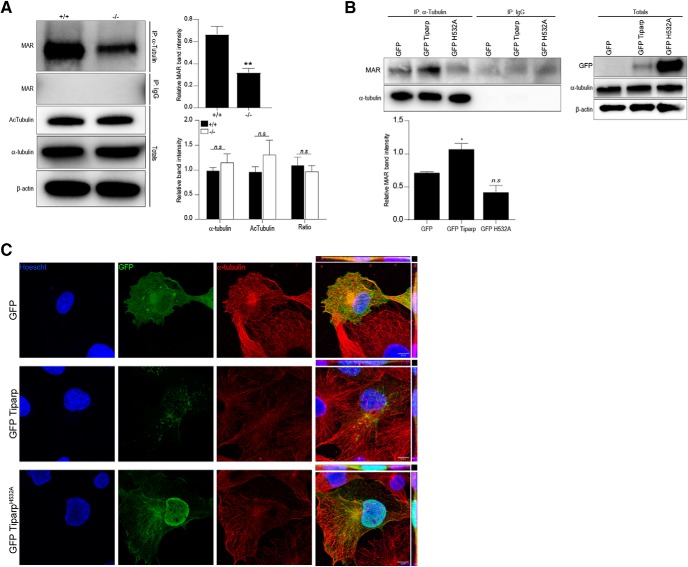
Tiparp ADP ribosylates α-tubulin. ***A***, Protein extracts from *Tiparp^+/+^*and *Tiparp^–/–^* mNSCs immunoprecipitation of α-tubulin followed by western blot against MAR. Total protein extracts were also immunoblotted against acetylated α-tubulin and α-tubulin. The right panel shows densitometry analysis of the relative intensity of the MAR band normalized to β-actin (top graph) and relative intensity of the acetylated α-tubulin, α-tubulin in the totals normalized to β-actin as well as the acetylated α-tubulin to α-tubulin ratio (ratio; bottom panel). ***B***, Cell extracts from COS-1 cells overexpressing GFP, GFP-Tiparp or the catalytic mutant GFP-Tiparp^H532A^ (labeled GFP-TiparpH532A) were immunoprecipitated with α-tubulin and immunoblotted against MAR and GFP to show co-precipitation of α-tubulin and Tiparp. ***C***, Cos-1 cells overexpressing GFP, GFP-Tiparp, or GFP-Tiparp^H532A^ and stained for α-tubulin. Data are mean ± SEM. The asterisk denotes significant differences between the wild type and the knock-out (*p < 0.05 and **p < 0.005) calculated with an unpaired *t* test (***A***, top panel), a two-way ANOVA (***A***, bottom panel), or an ordinary one-way ANOVA (***B***). Non-significance is denoted by n.s.

Since previous studies reported a link between α-tubulin and abnormal migration of cortical neurons (Keays et al., 2007; Li et al., 2012) and α-tubulin was recently reported to be ADP-ribosylated (Larsen et al., 2018), we explored the possibility that the defects in correct cell layering in the cortex of *Tiparp^–/–^* mice were due to altered regulation of α-tubulin which in turn led to abnormal migration. Western blot analysis showed a significant decrease in the levels of MAR of immunoprecipitated α-tubulin in *Tiparp^–/–^* cells compared with *Tiparp* ^+/+^ cells (Fig. 6*A*). We next transfected COS-1 cells with GFP-Tiparp or a catalytically deficient TIPARP, GFP-Tiparp^H532A^, and repeated the α-tubulin immunoprecipitation to verify that it the MAR was Tiparp-dependent. Transfection of GFP-Tiparp in COS-1 cells followed by α-tubulin immunoprecipitation showed an increase in MAR compared to cells transfected with GFP alone or with the GFP-Tiparp^H532A^ (Fig. 6*B*). The different levels of protein expression detected between wild-type Tiparp and the Tiparp^H532A^ catalytic mutant was due to the increased stability of the latter (MacPherson et al., 2013). To show co-localization of Tiparp with tubulin GFP, GFP-Tiparp and GFP-Tiparp^H532A^ were transfected in COS-1 cells and then immunostained with α-tubulin. Colocalization was observed in the cells bearing the GFP-Tiparp plasmid, but this was independent of Tiparp catalytic activity, since colocalization between α-tubulin and GFP-Tiparp^H532A^ was also observed (Fig. 6*C*). Taken together, these data suggest that Tiparp plays an important role to MAR of α-tubulin.

## Discussion

In this study, we demonstrate that Tiparp affects neural progenitor cell proliferation and migration, and its loss leads to aberrant organization of the mouse cortex. Although mild, the phenotype of the *Tiparp^–/–^* mice suggests that alterations in Tiparp expression or function could increase susceptibility to a wide range of both developmental and degenerative neurologic diseases.

We show that *Tiparp^–/–^* mice have less PNNs, which are structures responsible for synaptic stabilization. This observation agrees with a study which used LTP in rats as a cellular model of learning and memory that identified Tiparp as a highly upregulated gene and essential for the maintenance long-lasting LTP, suggesting a close association with plastic changes in the synaptic efficacy. This is achieved by the induction of structural alteration in the synaptic connections and their deep restructuring (Matsuo et al., 2000). However, whether this loss of PNNs is due to the loss of Tiparp is unknown but it does also suggest aberrant synaptic plasticity.

Furthermore, adult *Tiparp^–/–^* mice exhibited atypical laminar organization of the cortex with increased number of cells, and specifically neurons, in the upper layers of the cortex. Reduced staining for specific cortical layer markers was especially evident in Layer V of *Tiparp^–/–^* mice brains. Differentiation and migration of neuronal progenitors during the development of the neocortex is temporally controlled with the migration of cells to specific laminar layers occurring in an inside-out manner. Our findings suggest that Tiparp influences neuronal cell-class determination (layer destination of projection neurons) through regulation of the cell cycle and migration. Furthermore, the *Tiparp^–/–^* mice showed differences of GABAergic neuron distribution with lower amounts of calretinin and parvalbumin-positive interneurons, but higher numbers of calbindin-expressing neurons. The molecular mechanisms underlining this observation remain to be determined.

Ahr influences the migration and differentiation of neurons and specifically of GABAergic neurons (Huang et al., 2004; Qin and Powell-Coffman, 2004). Other studies report that activating the Ahr pathway in mice alters differentiation patterns of neural progenitors and leads to decreased numbers of non-GABAergic projection neurons and thinner deep neocortical layers due to the nuclear accumulation of cyclin-dependent kinase inhibitor p27^Kip1^ (Kolluri et al., 1999; Mitsuhashi et al., 2010). Moreover, the absence of Ahr expression compromises GABAergic neuron cell differentiation in the ventral telencephalon (Huang et al., 2004; Gohlke et al., 2009). Tiparp expression is induced by Ahr, which in turn regulates Ahr signaling as part of a negative feedback loop (MacPherson et al., 2013; Grimaldi et al., 2018). Therefore, loss of Tiparp could result in hyperactivation of Ahr signaling, which could partly explain our findings. Alternatively, Tiparp may function in the brain on activation of the Ahr pathway and subsequently target critical proteins in different pathways thus acting as an important downstream regulator of Ahr. Tiparp has been described as both a modulator and cross-pathway mediator of innate immunity. Constitutive Ahr signaling targets Tiparp which in turn mono-ADP ribosylates TBK1, thus suppressing the TBK1-mediated pathway that is essential for interferon induction and the innate immune response (Yamada et al., 2016).

However, Tiparp could also function independently of Ahr. Its expression can be induced by other nuclear receptors important in neuronal development such as glucocorticoid receptor and platelet-derived growth factor receptor (Schmahl et al., 2008; Chinenov et al., 2014). Indeed, *in utero* exposure to synthetic glucocorticoids results in aberrant distribution of neurons throughout the cortex, similar to what we report in this study (Tsiarli, 2014). The complex cytoarchitecture and laminar organization of the cerebral cortex are the result of several processes including neurogenesis, neuronal migration and differentiation. Cortical malformations often show abnormalities in all three processes, because genes involved in one of these processes often affect the other two (Manzini and Walsh, 2011). Cytoskeletal components, such as α-tubulin are key regulators of all aspects of neuronal differentiation have been associated with cortical malformations (Francis et al., 2006; Kerjan and Gleeson, 2007; Lian and Sheen, 2015). *Tuba1a* mutant mice have defects associated with cortical Layers II/III and IV and aberrant neuronal migration (Keays et al., 2007). A recent study reported that α-tubulin is mono-ADP ribosylated; however, the enzyme responsible for this posttranslational modification of α-tubulin was not reported (Larsen et al., 2018). Here, we provide evidence that Tiparp contributes to the MAR α-tubulin. Loss of Tiparp expression or its catalytically activity resulted in a significant decrease in mono-ADP-ribosylated α-tubulin. Since tubulin is regulated by many other mechanisms and other posttranslational modifications, the defects we observed were not as severe as those described in the *Tuba1a* mutant mice (Keays et al., 2007; Kumar et al., 2010; Magiera and Janke, 2014). Collectively, our results suggest that Tiparp plays a role in the development of the cerebral cortex. Further studies are needed to fully characterize its mechanism of action and to dissect the pathways involved in this regulation.

## Synthesis

Reviewing Editor: Robert Kalb, Northwestern University Decisions are customarily a result of the Reviewing Editor and the peer reviewers coming together and discussing their recommendations until a consensus is reached. When revisions are invited, a fact-based synthesis statement explaining their decision and outlining what is needed to prepare a revision will be listed below. The following reviewer(s) agreed to reveal their identity: Jonathan brent.

While there is much enthusiasm for this work there are a number of concerns, especially regarding data presentation and quantification. It would behoove the authors to directly address all of the concerns

Reviewer #1:

This paper entitled "Loss of Tiparp results in aberrant layering of the cerebral cortex" investigates the effect of loss TiPARP(PARP7) on the structure of the mouse brain. The authors observed an alteration in the distribution of the neurons in the cortex, specifically sub-types of GABAergic neurons; including a decrease in in parvalbumin containing neurons an increase in calbindin positive GABAergic neurons and a decrease in the PNNs. They observed no change in neuronal excitability in the cortex and in the Tiparp mouse compared to control. The authors hypothesized that Tiparp may regulate neuronal differentiation. To test this they isolated primary neurons from embryonic Tiparp and control mice and differentiated the cells into neurons, glia or oligodendrocytes. Real time PCR of these differentiated cell types revealed reduction in specific neuronal, glia and oligodendrocyte mRNAS, suggestive of impaired differentiation. The authors present data that suggest that proliferation of the neural stem cells are reduced and that this is because the cells are stuck in mitosis. Finally, the authors show that Tiparp coIPs with tubulin and that monoADP-ribosyaltion and acetylation of tubulin is reduced which the authors suggest may also explain why the neurons have reduced differentiation rates.

Uncovering a role of Tiparp in the development and function of the brain is of interest. The authors have thoughtfully examined the cell types that may potentially be altered in this mouse model. However, there are some major pieces missing from this story and at times the authors over interpret their data. I list my major and minor concerns below.

Major.

1. The authors state that the Tiparp mouse was generated as previously described suggesting that the authors themselves made this mouse model specifically for this study. if they did the authors should add data to show that Tiparp protein is reduced in this mouse model. The authors should also explain in the methods how this model was made.

2. The authors often do not explain their experiments fully, for example in the results section describing Figure 4A the authors claim that there is no significant difference between neural stem cells from normal and Tiparp mice but there is no explanation of what was looked at and why this was important. The authors also state in this section that no significant difference was observed in the mRNA expression or immunofluorescence staining in the 3 cell lineages, but go on to describe significant mRNA changes in Blbp and Ng2. This is a confusing set of sentences. The authors ignore the significant difference of TubIII mRNA, which is important because in Fig 6 they show data that suggests that posttranslational modification of Tubulin is reduced, perhaps this is because of reduced mRNA levels. The authors need to comment on this.

3. There is no quantification on the immunofluorescence in Figure 4 so the words "no significant difference" cannot be used to describe this data. The authors should remove this or add quantification of the data.

4. The proliferation studies are not appropriately described in the results section. Why was this performed, what was done to assess this and what does the data mean when there is less proliferation?

5. The authors should describe the significance of the cell cycle studies. Is that cells are failing to exit the cell cycle? Or they are becoming stuck in the cycle?

6. Scratch assay is nicely described in the legend but is not described as well in the results section.

7. The authors show that Tiparp and tubulin coIP, these data do not show that the protein directly interact. The authors should remove this wording from the abstract, article and legend. To show a direct interaction of the proteins would have to be purified and the coIP performed with the two isolated proteins (for example).

8. The authors observe a reduction in mono(ADP-ribosylation) and acetylation of tubulin in the Tiparp mouse this does not indicate that Tiparp is directly modifying tubulin in this assay. The authors need to be more precise in their choice of words.

9. The quantification of the coIPs in 6A is not present. The total levels of Tubulin should be measured against a second housekeeping protein to see if loss of Tiparp is reducing protein levels and that this may explain the reduction in ADP-ribosylation and acetylation.

10. The coIPs in 6A and 6B should be performed with an IgG control to demonstrate that Tiparp is not binding to the bead of IgG instead of Tubulin.

11. The immunofluorescence of Tiparp suggests the protein is mainly cytoplasmic but in the intro the authors state that the protein is mainly cytoplasmic. The authors should show the nuclear and GFP signals in separate panels and comment if there is a difference compared to the previous literature.

12. It also seems like inactive Tiparp interacts more strongly with tubulin. Is this the case? And if so why do the authors think this would happen.

Minor:

1. The link to Tiparp and GABAergic neurons was not made clear e.g.TCDD affects GABAergic neurons and activates Tiaprp so we looked at GABAergic neurons in the Tiparp -/- mouse.

2. The N needs to explained properly in all legends, when does it mean mouse, sections or cells?

3. Not all of the statistical methods are explained in the legends and often there are no details on the post-hoc test used. This needs to be clear in every legend. Also, if the data is not significant this needs to be in the bar charts so it is clear that statistics have been performed on the data and that there is no difference.

4. There are a lot of acronyms that are not explained in the text e.g. TCDD, mNSC, AHR.

5. The use of HA GFP in Fig 6B is confusing, and the full name should be used.

Reviewer #2

Positive(s) about the manuscript:

Overall, the work advances the field and the major claims of the manuscript are supported. Specifically, the cortex does appear to be disorganized with detailed analysis of GABAergic neurons showing perturbed subpopulations. Electrophysiologically the cortical cells appear to be pretty similar. They show that mNSCs in the mutant have similar gene expression although upon differentiation their are some slight differences among the three cell types. Generally, the differentiated cells look qualitatively similar as well. The mNCS appear to have abnormal proliferation and migration. There does appear to be decreased ADP ribosylation in the null mutant and the COS7 cells do appear to have colocalized TIPARP with tubulin.

Things to address which would add to the clarity of the presented work are listed below:

1. Extended table 1-1 appears to have been mislabelled. In this table the data is listed as 1A-1D, 3B and 3D. It is confusing as there are no bin numbers communicated in figure 1A- actually qPCR data related to specific tissues is in the figure. Furthermore, as written there is no statistical data communicated for figure 1E. Possibly they meant to label it figure 1B-E.

2. The images in Figure 2A are quite dim and I am concerned in the final version readers may have a hard time interpreting.

3. The manuscript argues that the PAX6/Nestin + cells in the right panel of figure 4A are qualitatively similar. This cells in the 2 images do not show similar density or morphology. Actually, they look quite different. It is possible that the 2 images are not representative of the actual findings or that there are differences. Possibly showing quantification of cell fluorescence in these images may detect a difference or support the authors claim that protein expression in the mNCS are similar.

4. The western data in figure 6 would be much easier to interpret with densitometry analysis. The authors suggest in the figure legend that this is provided in a lower panel in the provided figures suggesting that was done but it is not in any of the versions of the manuscript available to me. Overall, qualitatively the B-actin loading control in GFP-HA lane 6B looks like there was slightly less input than the other lanes. As the key argument of the figure is that there is slightly less ADP ribosylation upon transfection of TIPARP that is catalytically inactive, I would feel more confident in this western if the loading control looked more similar. Also possibly labelling it GFP-H532A would be less confusing as some may think you are referring to an HA tag.

## Author Response

Dear Reviewing Editor,

We would like to thank you and the reviewers for their constructive comments and for giving us the opportunity to revise our manuscript. We have addressed all of their concerns and we are confident that our revised version is significantly improved from its original form. We have carefully adhered to the instructions for re-submitting manuscripts with major revisions, as you outlined in your letter. You will find our detailed responses to reviewers' questions below.

- The sex of the species studied is not mentioned in the materials and methods section. Please mention “male”, “female”, “x males and x females”, “of either sex”.

We have amended this and we have now mentioned on page 4 line 104 that mice of either sex were used in the studies.

Reviewer #1:

Major.

1. The authors state that the Tiparp mouse was generated as previously described suggesting that the authors themselves made this mouse model specifically for this study. if they did the authors should add data to show that Tiparp protein is reduced in this mouse model. The authors should also explain in the methods how this model was made.

We have added a short description in the Materials & Methods section (page 3 lines 97-99) to summarize how the Tiparp knockout mice were generated. A full description of how the mice were generated has been previously published (Hutin, Tamblyn et al. 2018). That study also provided evidence showing reduced Tiparp levels by measuring mRNA expression and by genomic DNA sequencing. However, this was not done at the protein level due to unreliable commercially available anti- Tiparp antibodies that do not work well in our hands. We are trying to generate our own anti-Tiparp antibody, but we have thus far not been successful.

2. The authors often do not explain their experiments fully, for example in the results section describing Figure 4A the authors claim that there is no significant difference between neural stem cells from normal and Tiparp mice but there is no explanation of what was looked at and why this was important. The authors also state in this section that no significant difference was observed in the mRNA expression or immunofluorescence staining in the 3 cell lineages, but go on to describe significant mRNA changes in Blbp and Ng2. This is a confusing set of sentences. The authors ignore the significant difference of TubIII mRNA, which is important because in Fig 6 they show data that suggests that posttranslational modification of Tubulin is reduced, perhaps this is because of reduced mRNA levels. The authors need to comment on this.

We apologize for the confusion and lack of clarity. We have added a few lines to the main body of the manuscript (page 11 line 324-327) about our rationale for comparing the neural stem cell populations which was to see if the aberrant distribution of neurons in the cortex was due to aberrant differentiation, proliferation or migration of the developing neurons. We have also explained that the the two populations were compared by looking at the expression of basic neural stem cell markers at mRNA and/or protein level (page 11 lines 324-352). As suggested by the reviewer, we have corrected the confusing interpretation of the data in Figure 4B-D where we explain that there are no major differences in the ability of the Tiparp knockout cells to differentiate into the three lineages and only two genes seem to be affected, suggesting that Tiparp does not play a crucial role in differentiation of neural stem cells. But some differences were noted in the oligodendrocyte differentiation (page 11 line 341-343).

We have not commented on the different levels of TubIII (Tubb3) mRNA expression since only a trend for an increase in Tubb3 levels was observed in knockout compared with wildtype cells; however, this is not statistically significant. Furthermore, Tubb3 encodes for tubulin beta 3 class III, which is a β-tubulin isotype expressed specifically in terminally differentiated neurons and a common marker of neuronal differentiation whereas in figure 6 we investigate the post translational modification of α-tubulin, encoded but the gene Tuba1a.

3. There is no quantification on the immunofluorescence in Figure 4 so the words “no significant difference” cannot be used to describe this data. The authors should remove this or add quantification of the data. As suggested by the reviewer, we have changed the wording with regards to the immunofluorescence described in figure 4 to “there were no differences” (page 11 lines 324 and 352). We have not performed quantification of the data as we only wanted to show that both cell lines expressed the typical markers of the different lineages.

4. The proliferation studies are not appropriately described in the results section. Why was this performed, what was done to assess this and what does the data mean when there is less proliferation?

We have amended the text and added the following:

- On page 12 line 353-354 the reason why the experiment was performed: to see if aberrant layering was due to alterations in cell proliferation.

- In the results section on page 11 line 324-327 a brief sentence describing that the proliferation rate was assessed by a methylene blue assay.

- On page 12/13 line 380-383 a brief sentence describing the conclusions from this experiment and the cell cycle experiment where we state that “Although the molecular mechanisms underlying the aberrant distribution of neurons in the cortex remain elusive, our data suggest that cell cycle/proliferation and cell migration are two processes affected by the absence of Tiparp which could contribute to the observed phenotype”.

5. The authors should describe the significance of the cell cycle studies. Is that cells are failing to exit the cell cycle? Or they are becoming stuck in the cycle?

We have added a summary sentence on page 12 line 370-374 “Taken together these data suggest a mitotic defect with abnormal cell cycle progression resulting in a lower proliferation rate in the absence of Tiparp. Furthermore, our data are consistent with a previous report showing that knocking down TIPARP in HeLa cells results in higher phospho histone H3 staining and increased length of pre-metaphase mitosis (Vyas, Chesarone-Cataldo et al. 2013).” However, exactly what is causing the mitotic defect resulting in different proportion of cells in the various stages remains elusive. As we and others have observed a slower proliferation rate in many Tiparp knockout cell

lines it is something we are currently investigating in a more focused study.

6. Scratch assay is nicely described in the legend but is not described as well in the results section.

We have made this correction and added an explanation about how this assay was performed on page 12 line 376-377.

7. The authors show that Tiparp and tubulin coIP, these data do not show that the protein directly interact. The authors should remove this wording from the abstract, article and legend. To show a direct interaction of the proteins would have to be purified and the coIP performed with the two isolated proteins (for example).

We apologize for the statement and we have re-worded the abstract, significance statement, the main body of article and all figure legends to reflect this change. For example we have included the following statement “Tiparp is part of a complex that interacts with a-tubulin and plays a role in its mono-ADP-ribosylation.

8. The authors observe a reduction in mono(ADP-ribosylation) and acetylation of tubulin in the Tiparp mouse this does not indicate that Tiparp is directly modifying tubulin in this assay. The authors need to be more precise in their choice of words.

We thank the reviewer for this comment. We have modified the wording throughout the article to reflect the change. We have changed the description to “Tiparp plays a role in the mono-ADP-ribosylation of a-tubulin”.

9. The quantification of the coIPs in 6A is not present. The total levels of Tubulin should be measured against a second housekeeping protein to see if loss of Tiparp is reducing protein levels and that this may explain the reduction in ADP-ribosylation and acetylation.

We thank the reviewers for the comment and after having performed statistical analysis on our results we do not see a reduction of total levels of tubulin or acetylated tubulin in our data but there is a significant reduction in ADP-ribosylation in the absence of Tiparp. We have added densitometry analysis of our western blots for figure 6A and 6B and amended the text to reflect this.

10. The coIPs in 6A and 6B should be performed with an IgG control to demonstrate that Tiparp is not binding to the bead of IgG instead of Tubulin.

We have performed the requested experiments and have added them to figures 6A and 6B.

11. The immunofluorescence of Tiparp suggests the protein is mainly cytoplasmic but in the intro the authors state that the protein is mainly cytoplasmic. The authors should show the nuclear and GFP signals in separate panels and comment if there is a difference compared to the previous literature.

We have presented the data as requested by the reviewer in figure 6C and split the nuclear and GFP signals. We have also expanded and hopefully clarified the localization of Tiparp within the cell in the introduction (page 2 line 45-53). We added a reference which describes that TIPARP has a diffuse cytoplasmic localization during mitosis but forms nuclear foci during interphase in Hela cells (Vyas, 2013). We also reference Kozaki 2017 which reported that TIPARP translocates to the cytosol in response to cellular stressors.

12. It also seems like inactive Tiparp interacts more strongly with tubulin. Is this the case? And if so why do the authors think this would happen.

Inactive Tiparp is more stable, since auto-ribosylation activity of TIPARP contributes to its proteolytic degradation (MacPherson, Tamblyn et al. 2013). The increased stability of the catalytic mutant of TIPARP (TIPARP H532A) results in more TIPARP protein in the cell. We have transfected in the same amount of DNA and this is why we observe greater protein levels inactive Tiparp H532A compared with Tiparp WT in our experiments.

Minor:

1. The link to Tiparp and GABAergic neurons was not made clear e.g.TCDD affects GABAergic neurons and activates Tiaprp so we looked at GABAergic neurons in the Tiparp -/- mouse.

We have added a sentence that better links these two statements on page 10 line 294 (As TCDD has been previously shown to affect GABAergic neurons and TCDD activates Tiparp, we investigated whether loss of Tiparp had any effects on GABAergic neurons and their distribution.).

2. The N needs to explained properly in all legends, when does it mean mouse, sections or cells?

We have explained that the N number in figure 1, 2 and 5 refers to the number of mice.

3. Not all of the statistical methods are explained in the legends and often there are no details on the post-hoc test used. This needs to be clear in every legend. Also, if the data is not significant this needs to be in the bar charts so it is clear that statistics have been performed on the data and that there is no difference.

Descriptions of all statistical methods and post-hoc tests have been added to the legends and n.s. has been added to the bar charts where there were no statistically significant differences.

4. There are a lot of acronyms that are not explained in the text e.g. TCDD, mNSC, AHR.

We have described the acronyms the first time that they are used (TCDD: line 30; mNSC: line 104; AHR: line 38), but not in every subsequent section. All the acronyms are described and we have followed the eNeuro instructions to the best of our abilities. We have also refrained from describing acronyms that the publisher wishes not to be written in full (e.g. sIPSCs).

5. The use of HA GFP in Fig 6B is confusing, and the full name should be used.

Thank you for pointing out this potential confusing terminology. We have changed HA to H532A to avoid such confusion. Reviewer #2

Things to address which would add to the clarity of the presented work are listed below:

1. Extended table 1-1 appears to have been mislabelled. In this table the data is listed as 1A-1D, 3B and 3D. It is confusing as there are no bin numbers communicated in figure 1A- actually qPCR data related to specific tissues is in the figure. Furthermore, as written there is no statistical data communicated for figure 1E. Possibly they meant to label it figure 1B-E.

We thank the reviewer for pointing out this mistake and we have amended Extended Table 1-1 to reflect the correct data in the figures.

2. The images in Figure 2A are quite dim and I am concerned in the final version readers may have a hard time interpreting.

We have brightened the images in Figure 2A.

3. The manuscript argues that the SOX2/Nestin + cells in the right panel of figure 4A are qualitatively similar. This cells in the 2 images do not show similar density or morphology. Actually, they look quite different. It is possible that the 2 images are not representative of the actual findings or that there are differences. Possibly showing quantification of cell fluorescence in these images may detect a difference or support the authors claim that protein expression in the mNCS are similar.

We agree with the reviewer. We have repeated the experiments and taken more representative images where the cell density is comparable between the two cell lines. The similarities are more evident and there are no morphological differences between the lines. We did, however, observe that the Tiparp knockout cells tended to clump together and this could be related to the observed differences in cell migration but this is just an anecdotal observation. Regarding the quantification, we did not perform this as we wanted to show that both cells are able to express the typical markers of the different lineages.

4. The western data in figure 6 would be much easier to interpret with densitometry analysis. The authors suggest in the figure legend that this is provided in a lower panel in the provided figures suggesting that was done but it is not in any of the versions of the manuscript available to me. Overall, qualitatively the B-actin loading

control in GFP-HA lane 6B looks like there was slightly less input than the other lanes. As the key argument of the figure is that there is slightly less ADP ribosylation upon transfection of TIPARP that is catalytically inactive, I would feel more confident in this western if the loading control looked more similar. Also possibly labelling it GFP- H532A would be less confusing as some may think you are referring to an HA tag.

We have added densitometry analysis for figures 6A and 6B. We have also repeated the western blots to show more equal loading. We agree with the reviewer that the labelling of HA could be confusing and have changed in to GFP H532A.
