# Supplementary material for: Loss of Tiparp Results in Aberrant Layering of the Cerebral Cortex
Source: eNeuro. 2019 Nov 22;6(6):ENEURO.0239-19.2019. doi: 10.1523/ENEURO.0239-19.2019 (PMC6883171; doi:10.1523/ENEURO.0239-19.2019)
Supplement: Extended Data Figure 1-1 — U values for the Mann–Whitney tests in Figure 1. Download Figure 1-1, DOC file. [file sup_enu-eN-NWR-0239-19-s01.doc]

| **Figure** | **Bins** | **p value** | **Sum of ranks in +/+, -/-** | **Mann-Whitney U** |
| --- | --- | --- | --- | --- |
| **Figure 1 B** | Bin 1-5 | 0.6905 | 25, 30 | 10 |
|  | Bin 6-10 | 0.0317 | 17, 38 | 2 |
|  | Bin 11-15 | 0.0556 | 18, 37 | 3 |
|  | Bin 16-20 | 0.3095 | 33, 22 | 7 |
|  | Bin 21-25 | 0.8413 | 29, 26 | 11 |
|  | Bin 26-30 | 1 | 28 , 27 | 12 |
|  | Bin 31-35 | 0.0079 | 40, 15 | 0 |
|  | Bin 36-40 | 0.2222 | 34, 21 | 6 |
|  | Number of cells | 1 | 27, 28 | 12 |
| **Figure 1 C** | Bin 1-5 | 0.5476 | 31, 24 | 9 |
|  | Bin 6-10 | 0.0079 | 15, 40 | 0 |
|  | Bin 11-15 | 1 | 28, 27 | 12 |
|  | Bin 16-20 | 0.0079 | 40, 15 | 0 |
|  | Bin 21-25 | 0.0079 | 40, 15 | 0 |
|  | Bin 26-30 | 0.5476 | 31, 24 | 9 |
|  | Bin 31-35 | 0.0317 | 17, 38 | 2 |
|  | Bin 36-40 | 0.952 | 36, 19 | 4 |
|  | Number of cells | 0.7 | 9, 12 | 3 |
| **Figure 1 D** | Bin 1-5 | 0.4206 | 32, 23 | 8 |
|  | Bin 6-10 | 0.4206 | 23, 32 | 8 |
|  | Bin 11-15 | 1 | 28, 27 | 12 |
|  | Bin 16-20 | 0.0159 | 39, 16 | 1 |
|  | Bin 21-25 | 0.0556 | 18, 37 | 3 |
|  | Bin 26-30 | 0.0079 | 15, 40 | 0 |
|  | Bin 31-35 | 0.0119 | 15, 40 | 0 |
|  | Bin 36-40 | 0.01508 | 20, 35 | 5 |
| **Figure 1 E** | Bin 1-5 | 0.3095 | 33, 22 | 7 |
|  | Bin 6-10 | 0.2222 | 34, 21 | 6 |
|  | Bin 11-15 | 1 | 18, 27 | 12 |
|  | Bin 16-20 | 0.0159 | 16, 39 | 1 |
|  | Bin 21-25 | 0.0079 | 15, 40 | 0 |
|  | Bin 26-30 | 0.0079 | 15, 40 | 0 |
|  | Bin 31-35 | 0.0079 | 15, 40 | 0 |
|  | Bin 36-40 | 0.0317 | 17, 38 | 2 |
