# Supplementary material for: Loss of Tiparp Results in Aberrant Layering of the Cerebral Cortex
Source: eNeuro. 2019 Nov 22;6(6):ENEURO.0239-19.2019. doi: 10.1523/ENEURO.0239-19.2019 (PMC6883171; doi:10.1523/ENEURO.0239-19.2019)
Supplement: Extended Data Figure 4-1 — U values for the Mann–Whitney tests in Figure 4. Download Figure 4-1, DOC file. [file sup_enu-eN-NWR-0239-19-s02.doc]

| **Figure** | **Panel** | **p value** | **Sum of ranks in +/+, -/-** | **Mann-Whitney U** |
| --- | --- | --- | --- | --- |
| **Figure 4 A** | Sox2 | 0.2286 | 12, 16 | 2 |
|  | Pax6 | 0.2286 | 20, 8 | 2 |
|  | Nestin | 1 | 11, 10 | 4 |
| **Figure 4 E** | mNSCs vs. Neuronal | 0.0002 | 238, 87 | 9 |
|  | mNCSs vs. Astrocytic | 0.0021 | 92, 79 | 1 |
|  | mNCSs vs. Oligodendrocytic | 1 | 110, 26 | 19 |
