# Supplementary material for: Loss of Tiparp Results in Aberrant Layering of the Cerebral Cortex
Source: eNeuro. 2019 Nov 22;6(6):ENEURO.0239-19.2019. doi: 10.1523/ENEURO.0239-19.2019 (PMC6883171; doi:10.1523/ENEURO.0239-19.2019)
Supplement: Extended Data Figure 5-1 — U values for the Mann–Whitney tests in Figure 5. Download Figure 5-1, DOC file. [file sup_enu-eN-NWR-0239-19-s03.doc]

| **Figure** | **Panel** | **p value** | **Sum of ranks in +/+, -/-** | **Mann-Whitney U** |
| --- | --- | --- | --- | --- |
| **Figure 5 B** |  | 1 | 18, 18 | 8 |
| **Figure 5 D** | Middle panel | 0.0015 | 171, 609 | 66 |
